# Cadmium Monitoring at the Workplace: Effectiveness of a Combination of Air- and Biomonitoring

**DOI:** 10.3390/toxics11040354

**Published:** 2023-04-08

**Authors:** Noömi Lombaert, Mik Gilles, Violaine Verougstraete

**Affiliations:** 1 International Cadmium Association, 1150 Brussels, Belgium; 2 International Zinc Association, Reach Cadmium Consortium, 1150 Brussels, Belgium; 3 Eurometaux, 1150 Brussels, Belgium

**Keywords:** cadmium, occupational exposure, biomonitoring, air monitoring, risk management measures, regulatory decision-making, dose response, threshold

## Abstract

Inhalation exposure to cadmium at the workplace has been associated with an increased risk of lung cancer and non-cancer respiratory effects. To ensure levels of cadmium remain below effect levels, air quality is monitored and regulations specifying an air limit value are implemented. The EU Carcinogens and Mutagens Directive of 2019 recommended values for the inhalable fraction and the respirable fraction but the latter only for a transitional period. Cadmium exposure has also been associated with systemic effects, following its storage in the kidneys and due to its long half-life. The accumulation of cadmium occurs via different exposure routes and from different sources, including workplace dust and fumes, food, and smoking. Biomonitoring (in blood, urine) has been identified as the most appropriate method to follow up cumulative exposure and total cadmium body burden, as it conveniently reflects intakes by all routes. However, it is not systematically implemented. This paper has a double objective: first, proposing a possible limit value for the respirable fraction, using an approach integrating epidemiological data. Secondly, demonstrating that the implementation of both air and biological limit values is key to protecting workers’ health in occupational settings. The paper summarizes the current knowledge on cadmium health effects and how biomarkers reflect those. It presents an approach to derive a respirable value, using recent human data, and describes how the combination of air monitoring and biomonitoring is applied by the EU industry to protect the workforce. While a respirable fraction value helps protect workers against local respiratory adverse health effects, air monitoring alone is not sufficient to protect workers against systemic effects of cadmium. Therefore, complementary biomonitoring and the implementation of a biological limit value is recommended.

## 1. Introduction

Occupational exposure to cadmium (Cd) occurs in production and refining facilities (smelters), nickel-cadmium battery manufacture and recycling plants, pigment manufacture and formulation plants, cadmium alloy production, mechanical plating, zinc smelting, brazing with cadmium-containing silver alloy solders, and in polyvinyl chloride-producing factories. Cadmium and its compounds have been classified as carcinogenic to humans (e.g., by IARC; under the EU CLP) based on experimental and epidemiological studies that identified an increased incidence of lung cancer after prolonged inhalation of cadmium dust. Increased risks of prostate and kidney cancer have also been reported [1].

Following exposure to cadmium via the oral or inhalation routes, the cadmium is partially absorbed by the body and much of it is stored in the kidneys. There is also excretion of cadmium, which can be measured by analysing urine samples. The concentration of cadmium in urine correlates very well with the amount of cadmium stored in the kidneys. Therefore, urinary cadmium is considered as a good representation of absorbed cadmium. 

A binding occupational exposure limit value of 1 µg/m³ (inhalable fraction) was set for cadmium in 2019 under the EU Carcinogens and Mutagens Directive (Directive (EU) 2019/983) after a transition period of 8 years (until 11 July 2027). Within this transition period, the limit value for the inhalable fraction is 4 µg/m³, and in those Member States with an implemented biomonitoring system, a biological urinary limit value of 2 µg Cd/g creatinine, together with an air limit value of 4 µg/m³ respirable fraction, can be used. 

Our commentary seeks to provide a general overview of cadmium adverse health effects to support the plea that the derivation of occupational exposure limits requires identifying both the relevant cadmium health effects and appropriate indicators. To protect against local respiratory effects primarily occurring in the deep lung, we propose to use a respirable fraction value, which can be derived using the approach recently followed by the German BAuA (2021) [2] but integrating also recent epidemiological data. 

We also want to demonstrate, based on monitoring data collected from over 5000 workers in 40 EU cadmium plants over more than a decade, that the implementation of a combination of a biological limit and occupational air limit value is an effective approach to ensure a steady decrease of the cadmium body burden in exposed workers.

## 2. Health Effects of Cadmium of Relevance to the Workplace and Derivation of Exposure Risk Relationships (ERRs)

For cadmium, there are two types of exposure that need to be distinguished:

(1) Exposure and uptake of cadmium via all routes (inhalation, ingestion, skin) followed by its distribution via the blood to target organs (e.g., kidney), causing systemic effects. For cadmium, such exposure can be monitored through biomarkers of exposure such as Cd in urine (Cd-U).
(2) Direct exposure of organs ‘at port of entry’, causing local adverse health effects. Specifically for cadmium, this concerns the respiratory tract through inhalation. Hence the exposure associated with these effects can be best monitored through air monitoring of Cd (Cd-air).

Different exposure indicators can be used to reflect the health effects of cadmium:

*Cd air* [expressed in µg/m3].

Since inhalation is a major cadmium exposure pathway at the workplace, the worker’s exposure to cadmium is generally assessed by measurement of the total cadmium concentration in workplace air (static sampling) or by personal air sampling. 

The principle of most of the personal measurement methods consists in trapping the sample on a suitable filter by using a particle sampler for inhalable or respirable fractions. The cadmium compounds are extracted and further analysed using an appropriate technique. 

*Cd-U* [expressed in µg Cd/g creatinine] is a biomarker which reflects cumulative exposure of the worker (the biological half-life of cadmium in the human body is 10–30 years in muscles, kidney cortex and liver tissue [3,4,5]. Cd-U integrates exposure and subsequent absorption from both ingestion and inhalation. 

Cadmium in urine is generally accepted as a biomarker of cadmium body burden reflecting long-term accumulation in the event of occupational or high environmental exposures. Studies among industrial workers and populations with high environmental exposure to cadmium have demonstrated that Cd-U rises in parallel with kidney cortex (Cd-K) and remains elevated many years after cessation of exposure. Under normal environmental conditions, however, the significance of Cd-U as a non-invasive biomarker of cadmium body burden is less clear: Cd-U levels are influenced by several factors, including physiological variations related to normal (age, circadian rhythm) and stress conditions (physical stress, smoking) or silent (undercurrent) pathological conditions. All these factors are affecting kidney pathways and coexcretion patterns of renal functional biomarkers and cadmium itself. This coexcretion of cadmium and proteins adds uncertainty to the relationship between Cd-U and the body burden of cadmium at low Cd-U values [6,7].

*Cd-B* (Cd concentration in blood) [expressed in µg Cd/L whole blood] is a biomarker which is influenced both by recent exposure (over the past 3 months) and by cumulative exposure (integrated over 20 years), arising from both ingestion and inhalation. However, the variation of Cd-B over two consecutive dates, if less than a year apart, reflects recent exposure, and its sensitivity to recent exposure (both up and down) is quite high [8,9]. Cd-B can be used to detect an equipment dysfunction or a poor implementation of hygiene policies which happened over the past 3 months. However, it requires more frequent and invasive sampling and is difficult to assess for workers with raised Cd-U values. Therefore, it can only be used to complement urine monitoring.

### 2.1. Systemic Effects

#### 2.1.1. Identification of the Most Sensitive Organ(s) for Systemic Adverse Health Effects 

Chronic exposure to cadmium, both at the workplace and in the general environment, may result in several systemic effects. The best-documented effect is on the kidney function. This organ is considered as the most sensitive one to the systemic toxicity caused by cadmium.

Other effects frequently reported are bone demineralisation leading to an increased risk of bone fractures and ultimately Itai-itai disease, but these were mainly described in the general population. Associations of cadmium exposure with the risk of several cardiovascular diseases have been reported in the general population but have not been reported in occupational studies [10]. The causality of these associations at low levels of exposure (CdU ≤ 1 µg Cd/g creatinine) has been challenged [7]. 

#### 2.1.2. Kidney Effects 

In occupationally exposed subjects, the first manifestation of cadmium nephrotoxicity is usually a tubular dysfunction resulting in a reabsorption defect and, hence, an increased urinary excretion of low molecular weight (LMW) proteins such as α1-microglobulin, ß2-microglobulin (ß2 M) and/or retinol-binding protein (RBP), but also calcium and amino-acids [11,12,13,14,15,16,17]. A cadmium body burden corresponding to a urinary excretion (Cd-U) of 5–10 μg Cd/g creatinine constitutes a threshold at or above which these tubular effects have been observed (Lowest Observed Effect Level (LOEL)). Studies examining the dose–response relationship between Cd-U and renal effects in workers are summarised in Table 1.

Some of these cross-sectional studies may have underestimated the true LOEL because of the inclusion of aged workers with previously much higher exposures, having probably lost a significant portion of their kidney cadmium burden when the study was conducted, resulting in a left shift of the dose–response relationship [28].

Tubular changes observed above 5–10 μg Cd/g creatinine are generally irreversible [29,30], and the association with further renal alteration, including a reduction of the glomerular filtration rate (GFR) [24,31,32], supports the health significance of this threshold (LOAEL).

An effect on the glomerulus may also be observed in cadmium-exposed workers, as indicated by increased urinary excretion of high molecular weight (HMW) proteins including albumin, immunoglobulins G or transferrin, but usually at high Cd-U [16,23].

Bernard et al. concluded that cadmium exposure levels that will not cause adverse kidney health effects amongst occupational exposed workers are considered as not causing any other systemic health effects [33]. The kidney effects are considered as critical systemic effects of cadmium exposure and used by regulatory bodies to define the exposure limit for these types of effects [2,18,34]. 

In occupational settings, the Lowest Observed Adverse Effect Level (LOAEL) has been identified as 5 μg Cd/g creatinine. From several studies conducted in Europe [35,36,37], United States [38] and Asia [39], it appears that renal effects can be detected in the general population for Cd-U below 5 µg Cd/g creatinine and even from 2 µg Cd/g creatinine or below. This points towards a LOAEL of 2 μg/g creatinine in the general population.

Therefore, a threshold value of 2 µg Cd/g creatinine would be protective for workers against systemic adverse health effects.

### 2.2. Local Effects

#### 2.2.1. Non-Cancer Lung Effects

For the derivation of a NOAEC (No-Observed-Adverse-Effect Concentration) for non-cancer lung effects, the evaluation by the European Chemical Agency (ECHA) (Scientific report for evaluation of limit values for cadmium and its inorganic compounds at the workplace, 2020) [40] refers to the human data of Cortona et al. [41]. This is a well-performed study in a factory producing silver–cadmium–copper alloys for brazing with a total of 69 male workers exposed to cadmium fumes. 

The human data of Cortona et al. have shown that changes in residual volume of the lung occur for a cumulative exposure to cadmium oxide fumes of 500 μg Cd/m³ × years, corresponding to 40 years exposure at a level of 12.5 μg Cd/m³ (LOAEC) [41]. CdO fumes consist entirely of very fine particles which are considered as respirable, also by Nordberg et al. [5]. The risk assessment by Nordberg et al. agreed on the 500 µg/m³ × years as the LOAEC for respiratory effects [5]. Nordberg et al. stated that “None of the more recent studies has documented effects of cadmium at lower exposures that can be considered caused by cadmium and not by smoking”. Applying an extrapolation factor of three (LOAEC to NOAEC) [42] leads to a value of 4 μg Cd/m³, respirable fraction [5].

The NOAEC value for non-cancer lung effect therefore becomes 4 µg Cd/m³ (respirable fraction).

#### 2.2.2. Lung Cancer

**Figure 1:**
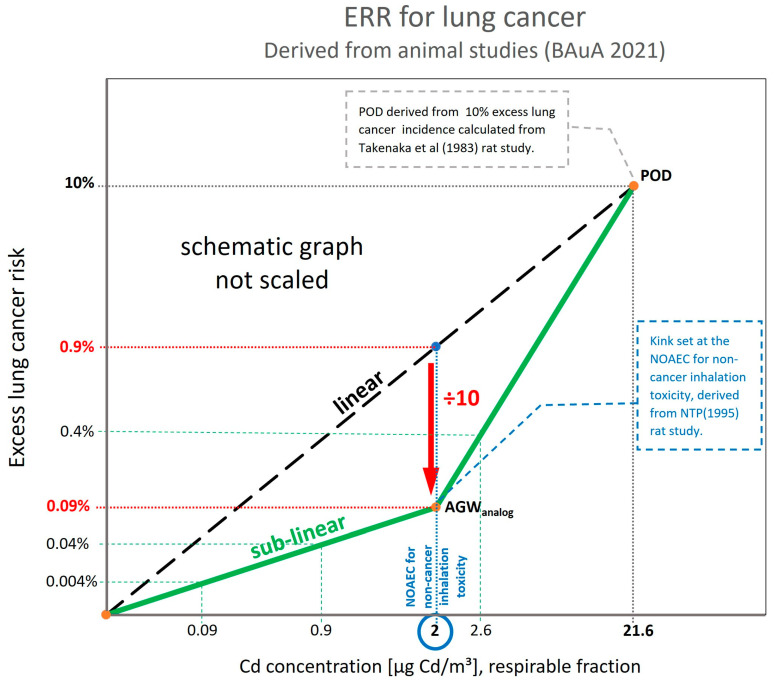
Exposure Risk Relationship (ERR): hockey-stick shape (lung cancer, cadmium), respirable fraction (adapted from BAuA (2021) [2]).

For deriving the ERR for lung cancer for cadmium, the BAuA (2021) [2] has updated the Technical Rules for Hazardous Substances–TRGS 910 document and integrated the recent science on mode of action for cadmium, which supports a practical threshold or mode-of-action-based threshold. Regarding the exposure risk relationship for lung cancer for cadmium, BAuA translated the non-quantified threshold for cancer effects in a hockey stick-shaped dose–response relationship (Figure 1). The starting point (Point Of Departure (POD)) of the hockey stick shape is the BMD10 value (exposure at which excess cancer risk is 10%). The kink in the hockey stick shape is positioned at the NOAEC for non-cancer inhalation risk. The hockey shape ERR is obtained by dividing the lung cancer excess rate at this NOAEC by a factor of 10.

It should, however, be noted that the BAuA assessment (2021) [2] used only animal data. The POD for lung cancer comes from the study by Takenaka et al. with cadmium chloride aerosols (MMAD = 0.55µm) [43]. The non-cancer effects come from the NTP sub-chronic inhalation rat study with cadmium oxide aerosol (MMAD = 1.1–1.6 μm) [44]. In this study, the NOAEC in the lungs was 0.025 mg CdO/m³ (= 22 µg Cd/m³) for rats. To recalculate the rat experimental exposure towards conditions representative of occupational settings, a factor of two was used to cover the subchronic-to-chronic extrapolation, a factor of two addressed the 8 h/day exposure at the workplace instead of the 6 h/day exposure in rats, and an interspecies factor of three was added; resulting in a NOAEC of 1.83 µg Cd/m³, respirable fraction. BAuA rounded this NOAEC and set the kink of the hockey stick at 2 µg Cd/m³ respirable fraction.

Calculating the excess lung cancer risk according to this hockey stick relationship results in risk levels of 4:10,000 at 0.9 µg Cd/m³ (respirable) and 4:1000 at 2.6 µg Cd/m³ (respirable). The relationships are shown schematically (not scaled) in Figure 1.

The ERR derived by the BAuA entails uncertainties related to the use of animal data. The study by Takenaka et al. [43] used to derive the BMD10 value (POD) presents severe weaknesses:

- The exposure regime of this study is quite unusual for an inhalation carcinogenicity bioassay. Exposure amounted to 23 h/day and 7 days/week for 18 months, which is far from conforming to standard OECD protocols (i.e., 6 h/day and 5 days/week for 24 months for rats). In addition, the post-exposure time of 13 months might increase the observed cancer cases due to spontaneously occurring tumours. There is some uncertainty in translating sensitivity of rats towards humans. Takenaka applied an interspecies assessment factor of three to translate this uncertainty. The overpredicted risk when using rat data was already mentioned by Thun et al. [45], who compared the lifetime risks of excess lung cancer predicted by the OSHA modelling of the Takenaka et al. bioassay [43] and from the Thun et al. epidemiological data [46]. Thun et al. demonstrated that “the risk as estimated from the Takenaka bioassay is substantially higher than that estimated from the human data, […] the risk is approximately one-tenth that of comparably exposed rats. The rat data overpredicts risk when compared to the observed increase in lung cancer mortality in epidemiologic studies of cadmium workers” [45] (pp. 637–638).
- Animal studies on other species revealed no increase in lung cancer risk, demonstrating the high degree of uncertainty created by using animal data [47].

When available, human data are preferred over animal data for deriving toxicity factors to avoid uncertainty due to interspecies differences [48]. For cadmium, good-quality data are available from occupational exposure studies and could be used to derive an ERR for lung cancer. BAuA (2021) [2] selected animal data because of the possible confounding effects of arsenic in human studies. However, these confounding effects, raised in the study of the Globe cadmium plant by Thun et al. [46], were analysed in further detail in later studies [45,49,50,51] and could be further deciphered. 

Haney [48] identified the study of Park et al. [51], which is the latest update of the Thun et al. cohort [46], as the most appropriate epidemiological study for the inhalation carcinogenic risk assessment of cadmium. The study of Haney [48] uses the lung cancer dose–response data from Park et al. [51] in its calculations of the updated inhalation unit risk factor (URF) for environmental exposure to cadmium. An URF had previously been derived by the U.S. Environmental Protection Agency [52] using the occupational study of Thun et al. [46] but was not updated till the revision of Haney [48]. 

Park et al. [51] re-analysed the cadmium smelter worker population from Thun et al. [46] exhibiting excess lung cancer, using more detailed work history information, a revised cadmium exposure matrix, a detailed retrospective exposure assessment for arsenic and updated mortality data (1940–2002). Additionally, the exposure assessment was refined by an analysis of personal protective equipment (PPE) with PPE protection factors, using in parallel air sampling (total fraction) and urinary cadmium concentration data. Park et al. [51] were able to isolate the possible confounding effect of arsenic and to derive a cadmium dose–response relationship by extracting data from the personal registries, relating the exact workplace monitoring data to the weekly activity of each worker. Additionally, biomonitoring data was available for both arsenic and cadmium and integrated into the assessment of exposure. The cancer risk from the exposure to arsenic for cadmium workers was independently calculated from arsenic cancer models. Based on reconstructed arsenic exposure profiles of all individual workers, 14 of the 36 total lung cancer deaths observed for the cohort were predicted to be attributable to cadmium exposure, while only five were predicted to be attributable to arsenic exposure. Interestingly, most of the arsenic-attributed deaths occurred in the four lower strata of cadmium cumulative exposure. This implies that the confounding effect by arsenic was rather limited and that the applied correction for this confounding effect is small.

These additional exposure details enabled Park et al. [51] to refine the initial study from Thun et al. [46] and address the shortcoming of having no clear split between cancer risks related to arsenic or to cadmium. With the new and much more detailed exposure analysis, Park et al. [51] could convincingly demonstrate that there was an excess lung cancer risk related to cadmium and also clearly quantify the excess lung cancer risks related to cadmium or arsenic exposure [48].

The possible confounding factor of arsenic being excluded, this makes it possible to use human data for deriving an ERR, following the BAuA protocol (2021) [2]. This requires defining a BDM10 for lung cancer (to set the point of departure (POD), and a NOAEL for non-cancer respiratory effects (to set the kink of the hockey stick shaped dose–response curve).

##### Deriving the BDM10 for Lung Cancer from Human Exposure Data

Park et al. [51] calculated the lung cancer risk (Standardized Mortality Ratio (SMR)) after adjustment for ethnicity and exposure to arsenic. These data were used by Haney [48] to derive an URF for the general population, using background risk data from Texas or from the US general population, a 5-year lagged cumulative exposure and a conversion factor of 2.8 (converting occupational concentrations into environmental concentrations for the general population). From this URF, the authors calculated a lifetime air concentration of 0.02 µg Cd/m³ corresponding to an excess risk of 10^−4^ general population). The risk can be easily converted back to the original occupational exposure by applying the same conversion factor of 2.8. The reconversion does not introduce any uncertainties. The incidence for occupational cancer is therefore 1:100 000 at a concentration of 0.02 µg Cd/m³ × 2.8 = 0.056 µg Cd/m³. **This corresponds to a BDM10 of 560 µg/m**³.

*Identifying the Monitored Air Fraction*

Considering that the target organs for lung cancer and the non-cancer lung effects are the lung alveoli, the respirable fraction, which is the fraction that can penetrate into the alveoli, is the one of highest relevance. Based on the calculations above, one further step is needed to derive the risk for a respirable fraction.

However, workplace air sampling at the Globe cadmium smelter (Thun-Park-Haney study) over the period 1920–1983 was not as advanced as today and did not refer to the respiratory fraction nor the inhalable fraction. Development of special IOM sampling heads to collect the inhalable fraction started only in 1986 by Mark and Vincent at the Institute of Occupational Medicine in Scotland [53]. When the inhalable IOM samplers became popular, comparative studies were done to assess the ratio between the “old total air”, like what was measured at the Globe smelter, and the inhalable fraction from the IOM sampler. The ratio for total/inhalable was reported to be in the range of 1:1.29 and 1:2.12 for cadmium in a lead smelter [54], a setting which is comparable to the Globe smelter. In contrast to what one might expect, the sampled “total” fraction is smaller than the inhalable fraction. A conservative value of 1:1.29 was withheld in the calculation below.

Correlations between inhalable and respirable fractions were also recently reported in a study where large datasets were collected from occupational settings in which both fractions were sampled [55]. When splitting the results by type of cadmium exposure, reasonably strong correlations were obtained. For an exposure type (soldering, casting) that compares well with the exposure at the Globe plant, a respirable/inhalable ratio of 1:2 was found [55].

With the above information on conversion factors, the measured cadmium concentrations at the Globe plant can be converted to the relevant respirable fraction that causes lung cancer.

With:

AIR(inh) = 1.29 × AIR(tot) and AIR(inh) = 2 × AIR(resp),

the correlation can be calculated as:

AIR(resp) = 1.29/2 × AIR(tot) = 0.65 AIR(tot)

Assuming a linear non-threshold effect, the excess cancer risk stemming from the Haney study [48] can therefore be recalculated as 10:100 (BMD 10) at a concentration of 0.65 × 560 µg Cd/m³ = **364 µg Cd/m³ (respirable fraction**). On the basis of a linear relationship, this can also be expressed as a risk of 1.1:1000 with a 4 µg Cd/m³ respirable fraction.

##### Refining the Sublinear Model from BAuA with Human Epidemiological Data

If the model is applied but now using the results from Haney [48] for the cancer POD and the NOAEC identified by Cortona et al. [41] for the non-cancer effects at 4 μg Cd/m³, there is a significant change in the excess lung cancer risk.

The excess lung cancer risk at the NOAEC of 4 µg Cd/m³ (respirable) calculated from the linear ERR becomes (10:100)/(364 µg/m³:4 µg/m³) = 1.1:1000.

**Figure 2:**
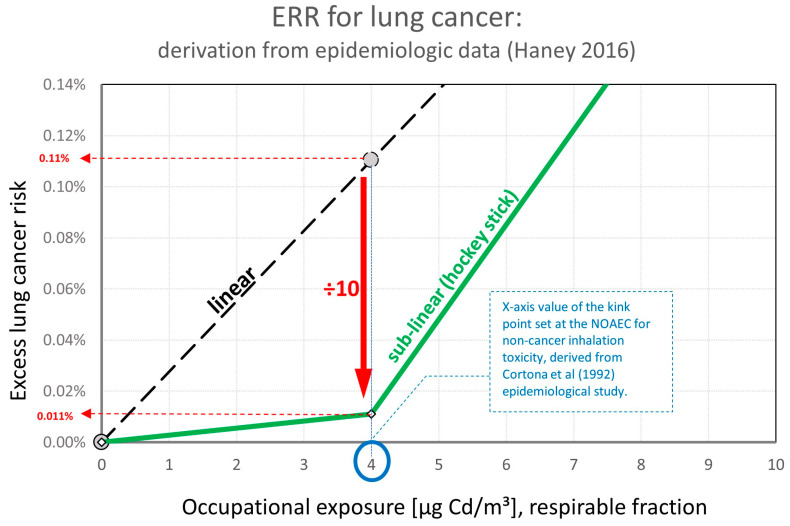
Exposure Risk Relationship (ERR): derivation based on epidemiological data Haney 2016 [48].

To introduce the sublinear “hockey stick” shape ERR, the excess lung cancer risk at NOAEC of 4 µg Cd/m³ (respirable) is reduced from 1.1:1000 to 1.1:10,000 (a factor of 10 reduction, as proposed by BAuA 2021 [2]) as schematically shown in Figure 2.

According to the calculated dose–response relationship derived from epidemiological studies, an exposure at levels of 4 µg Cd/m³ (respirable) is linked to an increased cancer risk of 1.1:10,000, which can be rounded to 1:10,000.

##### Conclusion on an ERR for Lung Cancer

Considering that:

- the NOAEC for non-cancer respiratory effect is 4 µg Cd/m³ (respirable fraction)
- the excess lung cancer risk at 4 µg/m³ (respirable fraction) is 1:10,000

It should be recommended to select and implement an OEL of 4 µg Cd/m³ (respirable fraction) to protect workers against respiratory adverse health effects. 

The respirable value is most representative for the expected respiratory effects (lung alveoli) and the derived value is based directly on human data, reducing the uncertainty of an extrapolation.

## 3. Decreasing Cadmium Body Burden: Combining Air- and Biomonitoring in the EU Cadmium Industry

Workplace air monitoring is an appropriate way to ensure no adverse health effects will occur in the respiratory tract (local effects). To protect against local respiratory effects occurring in the lung alveoli, monitoring the respiratory fraction is most relevant. In line with the EU SCOEL 2017 opinion, the International Cadmium Association (ICdA) recommends its members to respect a maximum respirable fraction of 4 µg Cd/m³. However, air monitoring alone is not fully effective in assuring worker protection against systemic effects of cadmium exposure, as it does not consider oral uptake of cadmium. Hence, complementary biomonitoring is done by industry to consider systemic adverse health effects following cadmium uptake by both inhalation and ingestion. ICdA recommends its members keep urinary cadmium of workers below 2 µg Cd/g creatinine, consistent with the SCOEL 2017 opinion.

The EU Industry Monitoring Observatory is a joint effort of the ICdA and its industry members, aimed at providing the workforce with a safe and healthy workplace. A ‘best practice’ ICdA Guidance was initially published in 1996 and is regularly updated. This Guidance is now in its 4th version, published in 2018 [56]. To monitor and measure progress with cadmium exposure, two major Monitoring Observatory programs were launched:

- starting in 2008, the collection of extensive occupational biomonitoring data on both cadmium in urine and cadmium in blood in Europe (OCdBIO)
- in 2014, the collection of monitoring data of cadmium in workplace air was added (OCdAIR)

### 3.1. Biomonitoring Observatory OCdBIO

In the Observatory of Cadmium Biomonitoring (OCdBIO), all participating companies’ occupational doctors provide anonymized biomonitoring data to the ICdA in full compliance with General Data Protection Regulation (GDPR). After collation, ICdA publishes a yearly consolidation together with an updated time trend series across the whole EU industry.

Starting in 2008 with 1800 workers in 15 plants, the OCdBIO observatory today covers annual reporting data on more than 5000 workers from 40 plants, in 12 countries. 

#### Biomonitoring Results: Urinary Cadmium

**Figure 3:**
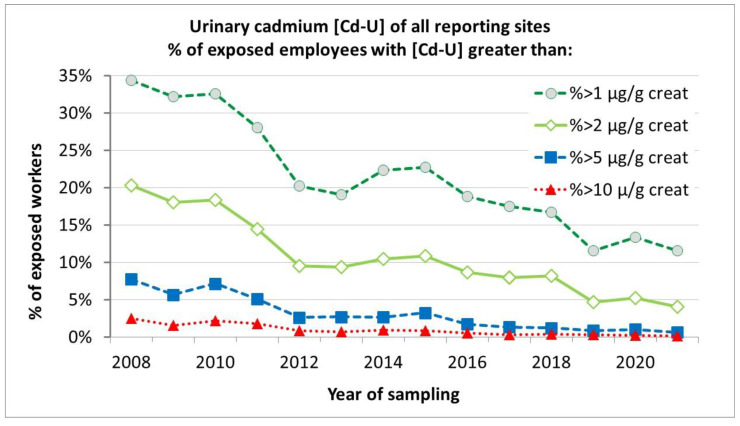
Reported urinary cadmium levels as % of exposed employees with Cd-U greater than x µg Cd/g creatinine from the period 2008–2021.

Figure 3 shows an overview of reported urinary cadmium levels from 2008 until 2021, illustrating the steady and significant decrease of urinary cadmium in occupationally exposed people. The share of workers with urinary cadmium > 2 µg/g creatinine has dropped from 20% in 2008 to only 4% in 2021.

It should be noted that clearing of historic accumulated cadmium from the human body is a very slow process. The decrease of urinary cadmium, even after a complete stop of cadmium exposure in this population, does not happen over a period of a few months but rather many years (10–30 years).

It should be noted that the ICdA Guidance [56] details best practices for plant and worker management in case cadmium levels increase and exceed 2 µg Cd/g creatinine. These include:

- An enhanced medical surveillance including regular measures of urinary cadmium to ensure workers protection.
- A detailed analysis of the related workplace environment along with an assessment of individual hygiene procedures implementation, including coaching by the occupational doctor.
- With occupational doctor consultation, removal from exposure if the CdU level of a worker exceeds 5 µg Cd/g creatinine.

The widespread implementation and monitoring of best practices across ICdA members over the years has allowed a demonstrable steady reduction of exposure, as witnessed by the evolution of urinary cadmium levels, which integrate accumulated exposure from both inhalation and ingestion.

### 3.2. Workplace Cadmium in Air Monitoring Observatory OCdAIR

#### 3.2.1. Data Reporting to ICdA and Fraction Measured

While biomonitoring allows the assessment of cadmium uptake of individuals, it is unable to identify the level of exposure in the respiratory tract; hence the need for additional monitoring of respirable cadmium. Contrary to biomonitoring, air monitoring is not done individually and permanently but is done by statistical sampling of cohorts. 

The ICdA started in 2014 the Observatory of Cadmium Air Exposure (OCdAIR), in which all participating companies report their workplace air exposure information following a very strict protocol. Note that the reported exposure concentrations are corrected for the use of personal respiratory protection.

In accordance with the SCOEL conclusions of 2010 and 2017, the fraction reported to ICdA is the respirable fraction, as defined by EN 481 ‘Workplace atmospheres; size fraction definitions for measurement of airborne particles’. Appropriate monitoring equipment and procedures compliant with EN ISO13137 to capture the respirable and/or inhalable fraction are selected by participating companies. Most plants measured and reported the respirable fraction, but some measured the inhalable fraction because national workplace limit values for cadmium in some countries referred to that fraction. For a few companies which report only data on the inhalable fraction, these are considered to encompass the respirable fraction and included as such in the OCdAIR database as conservative respirable data. From plants reporting both fractions, we see that inhalable and respirable fractions differ by a factor 5 to 10.

#### 3.2.2. Similar Exposure Groups (SEGs) and SEG Distribution

All participating plants are required to use the concept of Similar Exposure Groups (SEGs) which bring together workers with a similar exposure profile, consistent with workplace monitoring standard EN689 ‘Workplace exposure—Measurement of exposure by inhalation to chemical agents—Strategy for testing compliance with occupational exposure limit values’.

In 2021, as shown in Table 2, 33 plants, representing 211 different SEGs and 3607 workers, reported their workplace monitoring data on cadmium in air to the ICdA.

#### 3.2.3. Testing for Compliance

Compliance testing is conducted using (1) the geometric mean or (2) standard EN 689:2018 (70% confidence interval of the 90th percentile), of measured values for each SEG. Those values are calculated and compared with the 4 µg Cd/m^3^ respirable fraction which is to be considered in combination with the 2 µg Cd/g creatinine exposure biomarker.

##### Geometric Mean

If we consider the geometric mean as assessment criteria, excellent progress has been made, such that by 2021 there were only three Similar Exposure Groups (SEGs)–representing 24 workers–where the exposure limit of 4 µg Cd/m³ respirable was exceeded (see Table 3).

##### Monitoring Standard EN 689

Although the geometric mean value is a more representative indicator for the exposure and long-term accumulation of cadmium by workers, the revised monitoring standard EU EN689:2018 has now defined a different statistical indicator for general application in workplace exposure assessment, which is based on the 70% confidence interval of the 95th percentile. Since toxicity levels for cadmium in air have been derived based on a cumulative 40-year occupational exposure, the average exposure concentration would be more relevant to assess the exposure risk. Comparing average exposure values with the 70% confidence interval of the 95th percentile from the OCdAIR monitoring, the latter is typically lower by a factor of 10. Therefore, the EN689:2018 approach is more conservative by a factor of 10. The statistical analysis applied by most plants does not consider previous sampling results. Therefore, the simplified statistical approach considers compliance is met when all samples are below 10% of the OEL. This means that, in practice, exposure assessment following EN 689:2018 is more than 10 times more conservative than a geometric mean value. Considering how the OEL value is derived, the assessment of the cadmium occupational exposure by EN 689:2018 introduces an additional stringency (factor of 10).

When applying the more stringent statistical monitoring standard EN 689:2018, which most EU Member States implement, the limit value of 4 µg Cd/m³ (respirable fraction) is exceeded for 253 workers representing 7% of the exposed workers (see Table 4). 

The ICdA, via an annual Health and Safety meeting and regular review of its Guidance, assists the Members in statistical interpretation and provides valuable support on continuous improvement to further reduce occupational exposure. Continued efforts are done to develop and implement engineering solutions and procedures to comply with the target values in air. Where such solutions are not available, personal respiratory protection is used.

The data presented in Table 3 and Table 4 demonstrate that industry has been successful in increasing the number of workers for which workplace air is regularly monitored. By implementing this voluntary programme aligned to the EU SCOEL 2017 conclusions, the number of workers who belong to SEGs which comply with the OEL of 4 µg Cd/m^3^ (respirable fraction, geomean value) has increased to over 87%. Conversely, the number of reported workers for which the geometric mean value exceeds 4 µg Cd/m³ respirable fraction has dropped to 24, now representing only 0.66% of all workers.

### 3.3. General Conclusion on Cadmium Monitoring

- The 14th annual data collection of cadmium biomonitoring data from over 5000 exposed workers in 40 EU plants shows that urinary cadmium continues on the steadily decreasing trend line over the last 15 years, providing practical evidence that today’s cadmium exposure levels in industry are managed to remain below the effect levels.
- This improved status is achieved by continued reduction of cadmium levels in air at the workplace combined with more strict hygiene measures to minimize unintentional oral uptake of cadmium. It demonstrates that implementing a combination of a BLV of 2 µg Cd/g creatinine and an OEL of 4 µg Cd/m^3^ respirable fraction (as recommended by the EU SCOEL 2017 [18]) is an effective approach to ensure a steady decrease of cadmium body burden of exposed workers, ensuring that the risk of occurrence of chronic cadmium diseases by occupational exposure will be reduced to an insignificant level. Exposure management by air monitoring alone would not have allowed such a decreasing trend of cadmium body burden to be achieved.
- Furthermore, applying a statistical assessment for the air monitoring data, according to EN 689:2018, implicitly adds another safety factor of up to 10 to the cadmium chronic exposure risk. In practice, therefore, EN 689:2018 has the effect of assessing exposure by comparing to 10% of the OEL.

## 4. Discussion

### Cadmium Occupational Exposure Limit Recommendations

The European Parliament and the Council adopted a new limit value, at workplaces, of 1 µg/m^3^ for Cd inhalable fraction (Directive (EU) 2019/983) after a transitional period of 8 years. Within this transitional period, a value of 4 µg Cd/m^3^ is applied to the respirable fraction together with a biomonitoring system implemented with a biological limit value for Cd-U not exceeding 2 µg Cd/g creatinine. Switching from respirable to the inhalable fraction adds a further 5–10-fold reduction in the OEL value. Reducing the workplace limit from 4 µg Cd/m³ respirable fraction to 1 µg Cd/m³ inhalable fraction (as set in Directive (EU) 2019/983), means in practice that the SCOEL 2017 exposure limit value of 4 µg/m^3^ respirable fraction reduces by a factor of 20 to 40. This adds to the implicit safety factor of 10 from EN 689:2018. The downward exposure trend in the ICdA monitoring data shows there is no need for further lowering of exposure limit values as proposed by the EU Directive.

Workplace air monitoring is an appropriate way to ensure no adverse health effects will occur in the respiratory tract (local effects). However, to protect against local respiratory effects occurring in the lung alveoli, one should use the correct fraction for air monitoring. We propose to use a respirable fraction value of 0.004 mg/m³ derived from human data and following the recent BAuA sublinear approach (2021) [2], which supports a mode-of-action based threshold for cadmium. 

In addition, we recognize that air monitoring alone is not fully effective in assuring worker protection against systemic effects of cadmium exposure, as it does not consider oral uptake of cadmium. Occupational doctors have reported to ICdA that the lack of hygienic discipline has become a dominant contributor in cadmium uptake, which can be evidenced by an increase of urinary cadmium values of individuals monitored regularly. For example, based on biomonitoring we observed an increased uptake of cadmium among some young workers which was not identified through air monitoring. Further lowering of air limit values for cadmium will require the introduction of masks at the workplace or the use of masks with a higher protection factor. The lowering of the limit value might give a false sense of safety, as the obtained further reduced cadmium uptake by inhalation is far lower than the non-addressed oral intake by lack of hygienic discipline. Therefore, we support complementary biomonitoring and the setting of a BLV for protecting workers against systemic adverse health effects, which are related to the total cadmium body burden, because of cadmium uptake by inhalation and ingestion, rather than lowering the OEL value. Cadmium biomonitoring is far more implemented in industry than air monitoring and most often covers a wider group of workers in a plant. This is mainly due to the simplicity of biomonitoring. Urinary sampling is an uncomplicated and robust technique which allows to address the health status of individuals, whereas implementing and assessing personal air sampling is a complex operation which on top of that is subject to a higher degree of uncertainty as to the real exposure of an individual worker. Workers generally feel more assured by biomonitoring as it provides feedback from an independent third party on their personal health status. 

Furthermore, we consider biomonitoring a valuable tool in workplace exposure management to follow up on cumulative exposure and total cadmium body burden, which is a good indicator for setting thresholds for systemic effects. However, we do not agree with the recent ECHA conclusion that adverse effects occur at 1 µg/g creatinine [34], which contradicts a well-established conclusion that the LOAEL for workers is 5 µg/g creatinine and that a protective BLV should therefore be set at 2 µg/g creatinine [18]. Furthermore, the recently concluded five-year European HBM4EU project confirmed that in some Member States a significant fraction of the population shows background levels of Cd-U > 1 µg Cd/g creatinine. In this context, setting a BLV at 1 µg/g creatinine would create systemic discrimination against these populations. This EU-funded project published its overview on cadmium and concluded that a value of 2 µg Cd/g creatinine would be protective in the occupational setting [57]. 

The scientific literature confirms that kidney effects are still considered the critical effects of cadmium exposure and report a LOAEL of 2 µg Cd/g creatinine in the general population. In occupational settings, the LOAEL has been identified at 5 µg Cd/g creatinine. We, however, support a workplace BLV of 2 µg/g creatinine, to be on the conservative side.

Based on the state of knowledge and performed analysis, ICdA recommends a limit value of 4 µg Cd/m³ (respirable fraction) together with a biologic limit value of 2 µg Cd/g creatinine in urine.

## 5. Conclusions

The members of the ICdA have been implementing for more than a decade a strict set of measures to protect workers from exposure to cadmium. These measures are built on the SCOEL 2010 recommendation to implement both a BLV to protect workers against systemic toxicity of cadmium, mainly renal effects, and also an OEL necessary to protect workers against long-term local respiratory effects. This combined approach was confirmed by SCOEL in 2017 and acknowledged by the Commission in Directive 2019/983/EU.

The ICdA’s 14th annual EU sampling of over 5000 workers exposed to cadmium in 40 EU plants (sampling cadmium in air, in blood and in urine) demonstrates that implementing a combination of a BLV of 2 µg Cd/g creatinine and an OEL of 4 µg Cd/m^3^ respirable fraction (as recommended by SCOEL 2017) ensures a steady decrease of the cadmium body burden of exposed workers.

The value of 1 µg Cd/m^3^ inhalable fraction as currently set in the Directive (EU) 2019/983 goes much further, as it reduces the OEL value by a factor of 40. The downward exposure trend in the ICdA monitoring data shows there is no need for this lowering of the air exposure limit value as proposed by the EU Directive if accompanied by a biologic limit value in urine of 2 µg Cd/g creatinine.

The 14 years of practical experience have evidenced that the implementation of biomonitoring is now widely supported in the plants and their occupational doctors and that the limit values set forward by SCOEL are sufficiently low to reduce the risk of chronic cadmium diseases to an insignificant level.

## Figures and Tables

**Table 1:** Thresholds for renal effects in studies in occupational settings (adapted from SCOEL 2017-SCOEL/OPIN/336) [18].

Reference	Type of Industry	n	Glomerular Effects	Tubular Effects	Threshold (LO(A)EL)
[19]	Electronic workshop Ni-Cd battery factory Cd-producing plants	-	HMW proteins ß2 M-S Creatinine-S	ß2 M-U	Cd-U:10 μg/g creatinine (G and T)
[13]	Alkaline battery factory	102		ß2 M, RBP	Cd-U:10–15 μg/g creat
[20]	Cd smelter	53		ß2 M	Cd-U:13.3 μg/g creat
[21]	Secondary Cd users	26		ß2 M, RBP, NAG	Cd-U:5.6 μg/L
[22]	Cd pigment factory	29		ß2 M, NAG	Cd-U:<10 μg/g creat (NAG)
[23]	Non-ferrous smelter	58	albumin, transferrin, serum ß2 M	ß2 M, RBP, protein-1, NAG	Cd-U:10 μg/g creat (T; lower for G)
[24]	Zn-Cd smelter	108		GFR decline	Cd-U:10 μg/g creat
[25]	Cd alloy factory	105		ß2 M	Cd-U:10 μg/g creat
[16]	Zn-Cd smelter	37	albumin, transferrin	ß2 M, RBP and other markers	Cd-U:4 μg/g creat (G) Cd-U:10 μg/g creat (T)
[26]	Zn-Cd refinery	14		ß2 M	Cd-U:7 μg/g creat
[17]	Battery factory	561		ß2 M	Cd-U:1.5 μg/g creat (>60 y) Cd-U:5 μg/g creat (<60 y)
[27]	Battery factory	599		ß2 M	Cd-U:5.5–6.6 μg/g creat

G: glomerular effects, T: tubular effects, Cd-U: urinary cadmium, creat.: creatinine in urine.

**Table 2:** Numbers of plants, SEGs and workers in the ICdA cadmium in air workplace monitoring program “OCdAIR” for the period 2013–2021.

	2013	2014	2015	2016	2017	2018	2019	2020	2021
**# Plants**	12	22	20	16	30	25	31	33	33
**# SEGs**	67	142	131	124	162	165	204	216	211
**# Workers**	994	1548	1369	1278	2249	1857	3499	3662	3607

# = number of.

**Table 3:** ICdA cadmium in air workplace monitoring program “OCdAIR”: numbers of workers compliant (≤4 µg Cd/m^3^) based on geometric mean of measured values per SEG.

Assessment Using Geometric	Year of Sampling				
Mean	2017	2018	2019	2020	2021
Range [µg Cd/m³], respirable fraction	**Number of Workers in this Range**				
<4 µg Cd/m³ respirable	2169	1711	3241	3510	3437
non-conclusive ^a^	28	126	99	101	146
4 <=> 7	48	20	21	36	15
7 <=> 10					
>10	4		18	15	
Other non-compliant ^b^					9
Total	2249	1857	3379	3662	3607

SEG = Similar Exposure Group, ^a^ for workers in these workplaces; all samples are <4 µg Cd/m³, but the number of samples is insufficient (<3) for a valid statistical assessment. ^b^ For workers in these workplaces, at least one sample is > 4 µg Cd/m³, but the number of samples is insufficient (<3) for a more precise allocation to one of the three exceedance groups.

**Table 4:** ICdA cadmium in air workplace monitoring program “OCdAIR”: numbers of workers compliant (≤4 µg Cd/m^3^) using assessment criteria described in EN 689:2018.

Assessment According EN689	Year of Sampling				
	2017	2018	2019	2020	2021
Range [µg Cd/m³], respirable fraction	**Number of Workers in this Range**				
<4 µg Cd/m³ respirable	1441	852	2393	2476	2493
non-conclusive ^a^	517	521	553	698	861
4 <=> 7	158	147	124	65	34
7 <=> 10	41	99	67	29	35
>10	92	166	184	311	146
Other non-compliant ^b^		72	58	83	38
Total	2249	1857	3379	3662	3607

Note: The statistical exposure assessment described in the workplace monitoring standard EN 689:2018 assigns each exposed worker to a SEG and considers the 70% confidence interval of the 90th percentile. SEG = Similar Exposure Group. ^a^ For workers in these workplaces, all samples are <4 µg/m³, but there is an insufficient number of samples for the EN 689:2018 statistical assessment to conclude. ^b^ For workers in these workplaces, at least one sample is >4 µg Cd/m³, but the number of samples is insufficient (<3) for a more precise allocation to one of the three exceedance groups.

## Data Availability

Not applicable.
